# Whole exome sequencing and cluster analysis reveal that *EPB41L4A* mutation may trigger tooth agenesis

**DOI:** 10.1371/journal.pone.0357667

**Published:** 2026-09-09

**Authors:** Tian-Qi Li, Qin Zhang, Hui-Juan Wang, Zhao-Feng Ma, Bing-Yu Lu, Ke-Gui Hou, Xiang-Yu Zhang

**Affiliations:** 1 Beijing Shunyi Hospital, Beijing, China; 2 School and Hospital of Stomatology, Tianjin Medical University, Tianjin, China; Nigerian Institute of Medical Research, NIGERIA

## Abstract

**Objective:**

To detect and analyze the correlation between commonly mutated genes and known genes associated with tooth agenesis in patients with non-syndromic tooth agenesis. The aim is to explore new genes that may be associated with tooth agenesis, to provide a genetic reference for its prevention as well as for the clinical diagnosis and treatment of tooth agenesis.

**Methods:**

Genomic DNA was extracted from the peripheral blood of 18 congenitally edentulous subjects, and related gene mutations were identified by whole-exome sequencing. The genes related to maxillofacial development and the known pathogenic gene sequences of congenital tooth agenesis were selected for local alignment analysis of pairwise sequences, and the metric relationship of related sequences was determined. Hierarchical and fuzzy clustering methods were used for cluster analysis.

**Results:**

Hierarchical clustering and fuzzy clusterings yielded consistent results. The *EPB41L4A* gene clustered with a large number of well-known and well-defined genes associated with tooth agenesis. From the perspective of cluster analysis, it can be inferred that the genes clustered together generally have similar functions.

**Conclusion:**

*EPB41L4A*, which is involved in the Wnt pathway, may be a candidate gene warranting further investigation.

## 1. Introduction

Tooth agenesis is the most common genetic developmental disorder in dental clinics, characterized by varying degrees of congenital tooth loss [1]. Apart from total edentulism, the highest prevalence of tooth agenesis, based on population and geographic distribution, is found in Africa (13.4%), followed by Europe (7%), Asia (6.3%), Australia (6.3%), North America (5.0%), and Latin America and the Caribbean (4.4%). In the Chinese population, the prevalence of congenitally missing teeth in Asia ranges from 6.0% to 6.9% [2,3]. Congenitally missing teeth can lead to ectopia of neighboring teeth, resulting in alveolar bone loss and malocclusion [4]. From a modern medical perspective, missing teeth not only affect outward appearance but also create difficulties with eating, pronunciation, facial expression, and facial aesthetics. Ultimately, this condition has a significant socio-economic impact, not only on the individual’s quality of life but also on the family as a whole [5].

Although several potential and identified environmental factors have been shown to influence tooth agenesis, genetic defects play a crucial role in etiology [6]. To date, mutations in genes such as AXIN2 (OMIM*604025) [7], EDA (OMIM*300451) [8], MSX1 (OMIM*142983) [9], PAX9 (OMIM*167416) [10], and WNT10A (OMIM*606268) [11] have been found to cause congenital tooth agenesis. These genes are involved in the early regulation of tooth development, primarily through the NF-κB and Wnt signaling pathways. However, basic research on the genetic etiology of tooth agenesis remains limited, and mutations in these genes have only been detected only in a subset of affected individuals, suggesting that there may be other unidentified genetic defects contributing to the condition [8].

Cluster analysis is the process of partitioning a large dataset into multiple data categories, where subsets within the same category share certain characteristics, with a high degree of similarity among the objects in the same category and a high degree of dissimilarity between different categories. The similarity between different objects is assessed using the concept of distance metric between objects, and the distance metric depends on the specific problem being addressed [12]. It is an important data mining tool and an algorithm for separating data of similar nature [13]. In many areas of biomedical research, clustering has been increasingly applied in recent years to analyze the growing volume of data [14].

In this study, we performed whole-exome sequencing (WES) on 18 patients with tooth agenesis and found that 6 of them did not have a clear causative gene. The six patients were screened for mutations associated with maxillofacial development, and their genes were clustered with known and identified tooth agenesis genes. Two different methods, hierarchical clustering, and fuzzy clustering, were used for cluster analysis to resolve the differences between the clustering results. Based on the clustering results, the potential association of the unreported *EPB41L4A* gene with tooth agenesis was preliminarily explored, providing a possible genetic reference for tooth agenesis.

## 2. Subjects and methods

### 2.1. Subjects and informed consent

**Fig 1:**
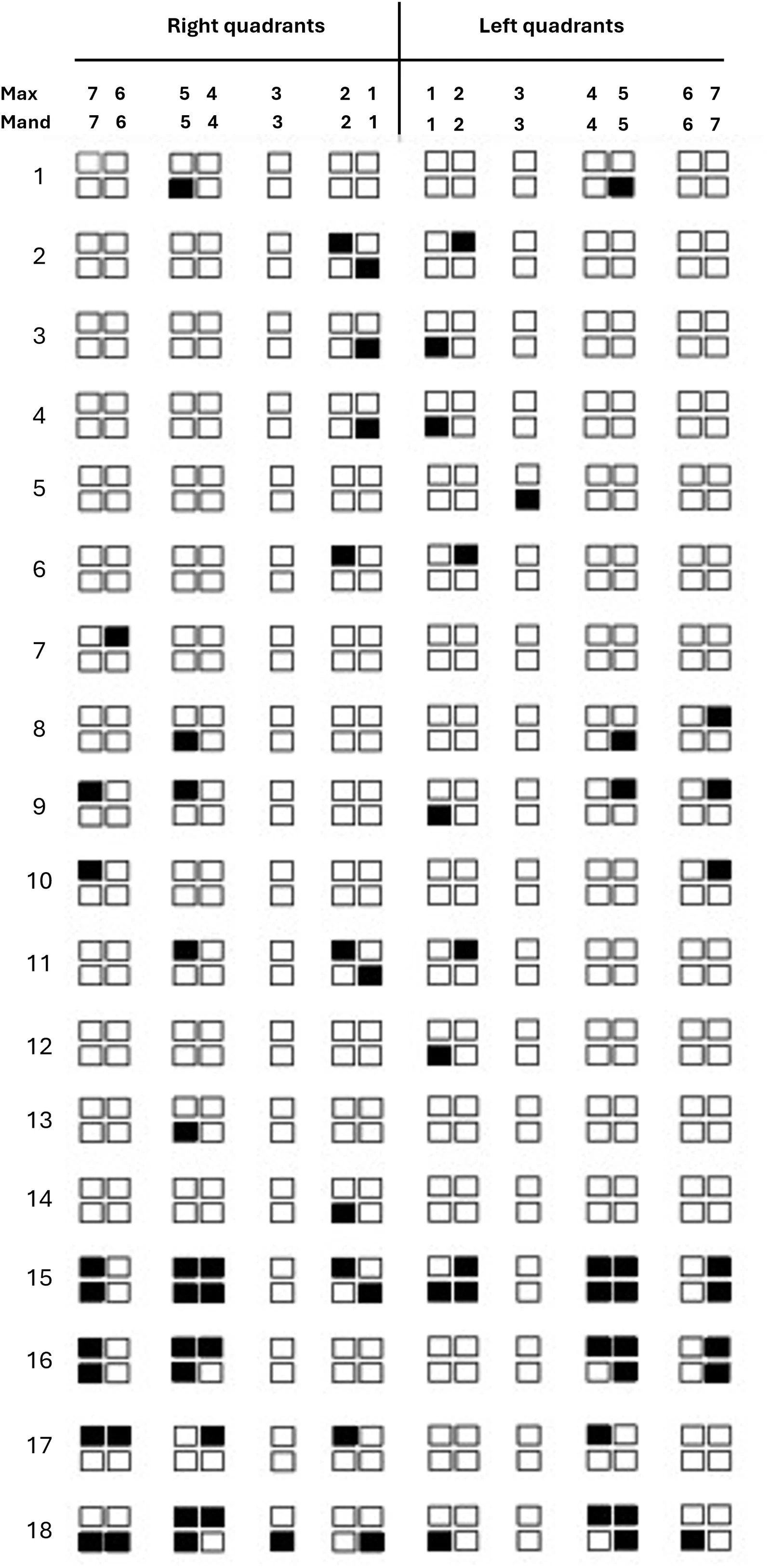
Tooth missing status of 18 subjects (participants 1-6 did not have a clear causative gene). The black solid indicates the missing tooth positions of the study subjects.

The study was approved by the Ethics Committee of Tianjin Medical University, and 18 subjects provided written informed consent (10/23/2019— 01/24/2024). All subjects were patients from the pediatric dentistry department, initially diagnosed with congenitally missing teeth (Fig 1). Panoramic radiographs were performed on 18 subjects to confirm the diagnosis of oligodontia. Two operators examined all patients using the same procedure.

### 2.2. DNA extraction

Genomic DNA was isolated from peripheral blood using QIAmp DNA Blood Midi Kit. The concentration and purity of the DNA samples were accurately quantified using the Qubit DNA Analysis Kit in Qubit®2.0 Fluorometer.

### 2.3. Whole exome sequencing

WES was performed by NovoGene Bioinformatics. Exon sequences were efficiently enriched using a liquid capture system, followed by capture with streptavidin magnetic beads and amplified by real-time PCR (3 nM). Finally, the DNA library was sequenced using an Illumina HiSeq 4000 sequencer.

The data filtering strategy was as follows:(1) Variants with a minor allele frequency (MAF) >0.01 in the 1000 Genomes Project (1000G, http://www.1000genomes.org) was excluded. (2) Single nucleotide polymorphisms (SNPs) with MAF > 0.01 were also excluded. (3) The remaining data were screened for genes related to missing teeth. (4) Bioinformatics analyses were performed on the remaining variants.

### 2.4. Mutation analysis and screening

Based on the WES results, we analyzed the mutated genes of 18 participants and found that mutations in the known genes that definitively cause tooth agenesis could be found in 12 participants (7–18): *P7-WNT10A; P8-AXIN2; P9-WNT10A; P10-WNT10A; P11-FGFR1* [15], among others. The remaining 6 participants (1–6) did not exhibit clear mutations responsible for their tooth agenesis. However, participants 1–6 shared 10 mutations in genes known to be associated with maxillofacial development: *CHD3* [16], *EPB41L4A* [17]*, PRDM2* [18]*, COL11A2* [19]*, BPTF* [20]*, EBF3* [21], *IQSEC1* [22], *ITPR3* [23], *TTN* [24], and *WDR33* [25].

### 2.5. Access to data

In the study of biological sequences related to tooth agenesis, this work focuses on mRNA‑sequence analysis. On the one hand, nucleic‑acid‑sequence analysis is fundamental and acts as the initial step for biological‑sequence research. On the other hand, mRNA can affect protein coding and translation, and sequence‑based analysis of mRNA lays a preliminary basis for subsequent exploration of protein structure and function [26].

To obtain nucleotide sequences of genes associated with tooth agenesis, we searched the nucleotide database maintained by the National Center for Biotechnology Information (NCBI). The keyword “tooth agenesis” was adopted, and “Nucleotide” was selected for the database category. A total of 29 mRNA sequences related to tooth‑agenesis‑associated genes were retrieved in March 2024. Finally, 20 available human‑derived mRNA sequences were screened out. Their NCBI accession numbers are listed below: A = {NM_002448, NM_022336, NM_022469, NM_001372076, NM_004655, NM_144991, NM_147127, NM_003394, NM_001347916, NM_025216, NM_006963, NM_001199687, NM_032045, NM_020789, NM_001005612, NM_018153, NM_001130144, NM_007055, NM_207111, NM_004892}.Following the same procedure, accession numbers for ten well‑known maxillofacial‑development‑related genes from Subjects 1‑6 were obtained from the database, as shown below: B = {NM_001005273, NM_022140, NM_015866, NM_080679, NM_004459, NM_001375392, NM_014869, NM_002224, NM_133379, NM_001006623}.

### 2.6. Determination of the distance matrix

For the above-obtained sequence datasets A and B are given variable names A = (X1, X2......X20), B = (X21, X22......X30), i.e.,: X1 = NM_002448, X2 = NM_022336,  ......, X20 = NM_004892, X21 = NM_001005273, X22 = NM_022140,  ......X30 = NM_001006623, and each variable name corresponds as in Table 1.

**Table 1:** Diagram of variable names.

Variable	Gene	Variable	Gene	Variable	Gene
X1	Msx1	X11	Znf22	X21	CHD3
X2	Edar	X12	Eda2r	X22	EPB41L4A
X3	Grem2	X13	Kremen1	X23	PRDM2
X4	Pax9	X14	Igsf9	X24	COL11A2
X5	Axin2	X15	Eda	X25	BPTF
X6	Tspear	X16	ANTXR1	X26	EBF3
X7	Evc2	X17	LTBP3	X27	IQSEC1
X8	Wnt10b	X18	POLR3A	X28	ITPR3
X9	Bmp4	X19	RNF216	X29	TTN
X10	Wnt10a	X20	SEC22B	X30	WDR33

**Fig 2:**
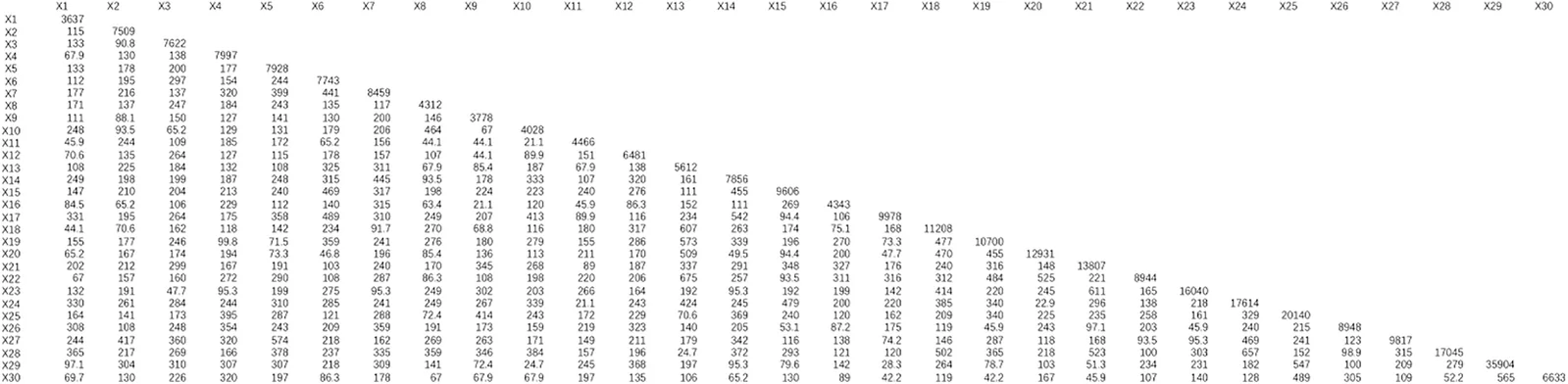
Score matrix X.

Apply the bioinformatics analysis tool BLAST to carry out the local comparison of two sequences (blastn suite, whose URL is https://blast.ncbi.nlm.nih.gov/Blast.cg) operation and analysis, and from this, we can get the score matrix X of sequence comparison: X= (X_ij_)_30×30_ (i,j = 1, 2,  ..., 30) is shown in Fig 2.

### 2.7. Data analysis

Two clustering methods are used: hierarchical clustering and fuzzy clustering. Hierarchical clustering begins by assuming that each individual data point forms a class and then iterates by clustering the most similar data points together. This iterative process reduces the number of classes with each step until all variables are consolidated into a single class. One of the research objectives of using this method in this study is to try to support the clustering results on the mutual synergy between biological sequences. The fuzzy clustering specifies that the sum of the affiliation of each sample to all clusters is 1, and each sample is given an affiliation function belonging to each cluster. The samples are categorized by the size of the affiliation value. The fuzzy clustering analysis in this study uses fuzzy relationships between biological sequences as a similarity measure to measure the affinity between biological sequences and classify them accordingly. This method involves constructing a fuzzy equivalence relationship matrix based on the similarity measure of the biological sequences under study and determining their classification according to the principle of maximum membership [27]. Since the correlations between biological sequences have a certain degree of ambiguity, the fuzzy clustering of classification in this study is more effective in analyzing and dealing with biological sequences with ambiguous phenomena in order to obtain more reasonable and reliable conclusions.

#### 2.7.1. Hierarchical Cluster.

**Fig 3:**
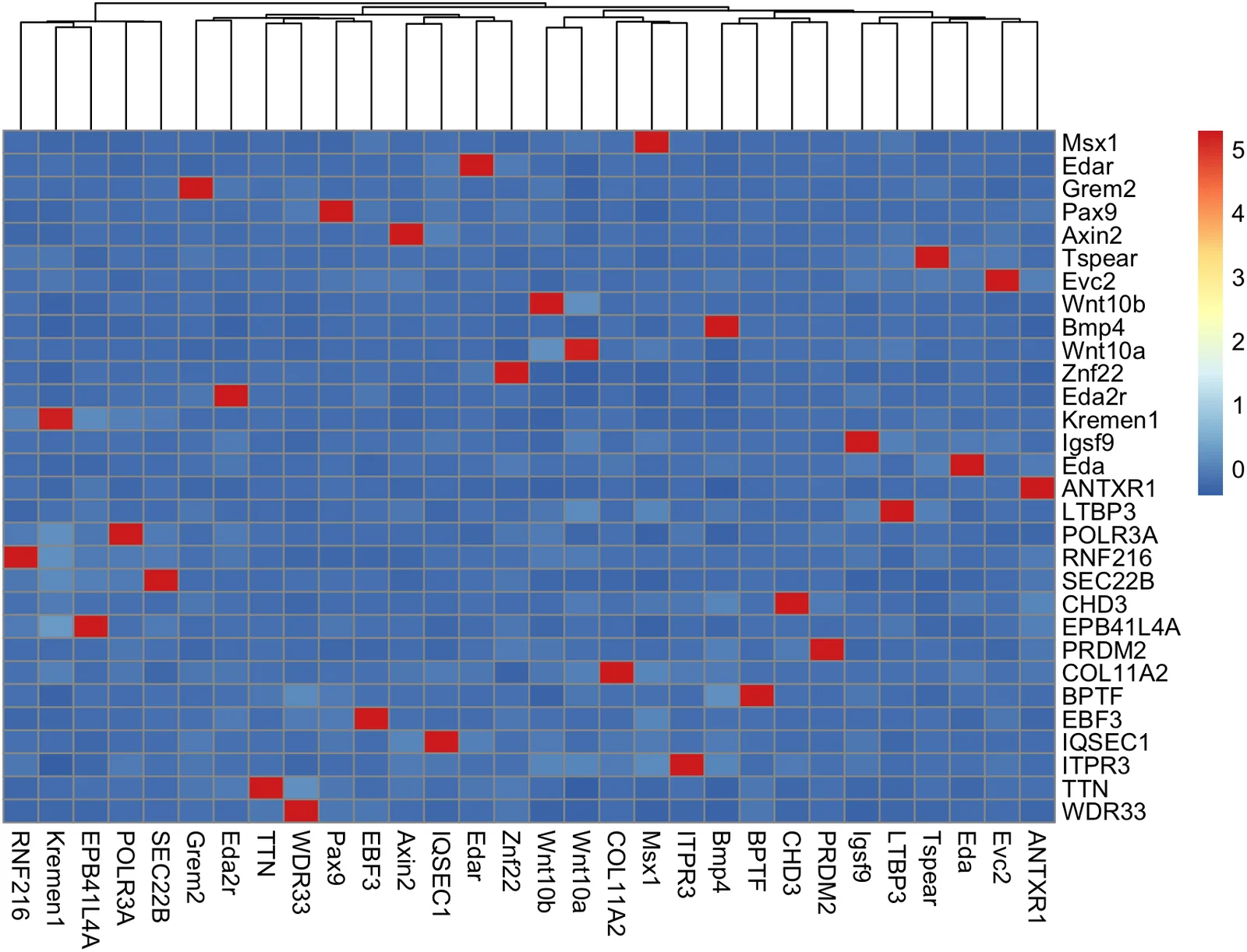
Hierarchical clustering results.

The Euclidean distance formula is used for the above comparison score matrix X. The distance relationship between the objects in the sequence is defined and calculated, and after normalization, hierarchical clustering is carried out using the ward method, resulting in the following hierarchical clustering diagram (Fig 3).

#### 2.7.2. Fuzzy Cluster.

**Fig 4:**
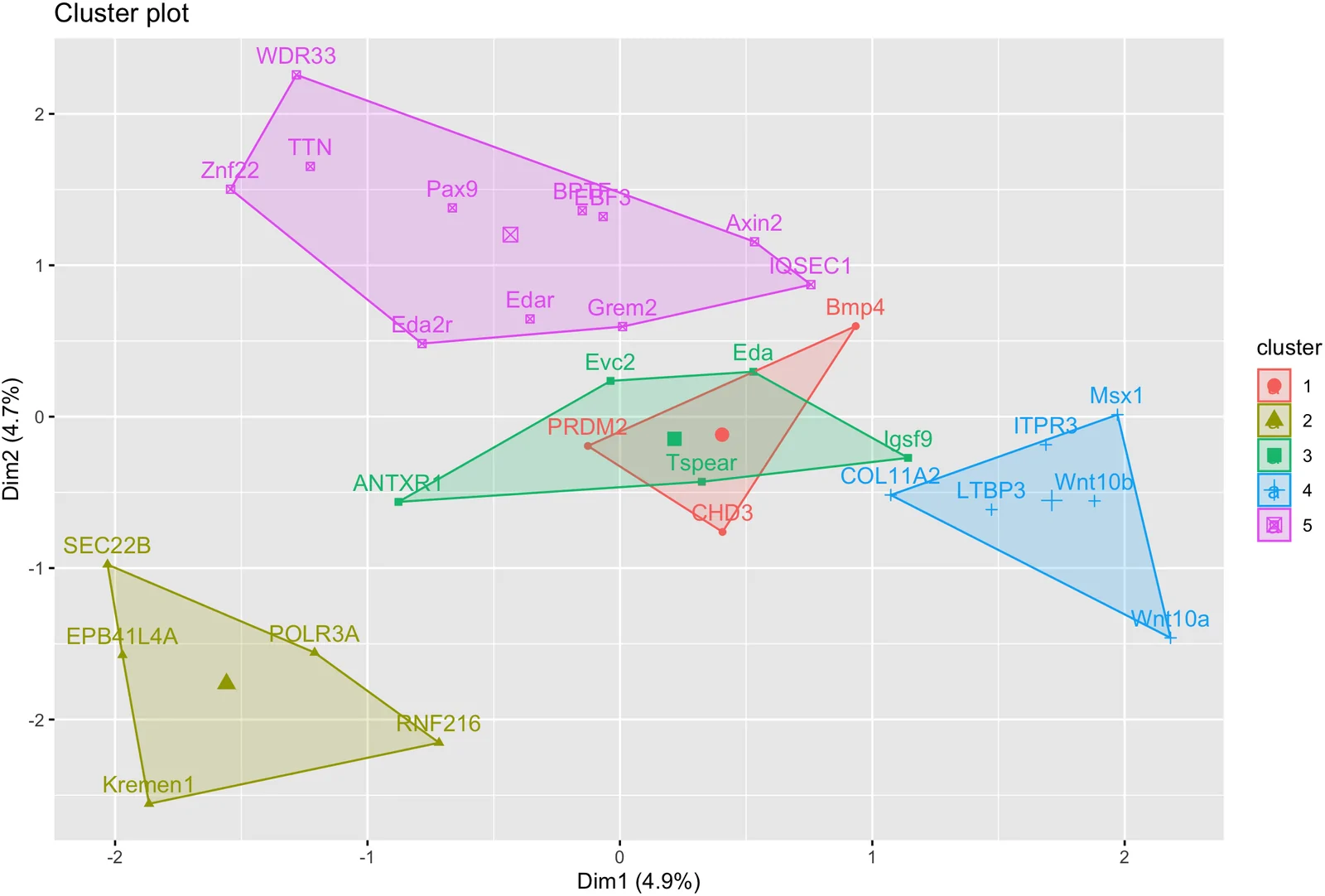
Fuzzy clustering results.

For the score matrix X= (X_ij_)_30×30_ (i,j = 1, 2,  ..., 30) of sequence comparison in the above dataset, the fuzzy similarity matrix R = (r_ij_)_30×30_ is obtained, and fuzzy cluster analysis is carried out using K-mean method, which results in the following fuzzy clustering diagram (Fig 4).

## 3. Results

The above two clustering result plots (Fig 3 and 4) show that both clustering methods grouped the sequences Kremen1, POLR3A, RNF216, SEC22B, and *EPB41L4A* into one class, where the sequences Kremen1, POLR3A, RNF216, and SEC22B are genes explicitly associated with congenital tooth loss in the database. The biological sequences clustered into one group in the cluster analysis have similar functions and similar properties [12], from this, it can be further inferred that: *EPB41L4A* is a plausible candidate gene that may contribute to tooth agenesis.

## 4. Discussion

This study aimed to identify genetic variants potentially contributing to tooth agenesis in 6 of 18 patients with tooth agenesis with an unclear etiology; 5 of the 6 patients had missing anterior teeth and 1 had missing premolar teeth. Through cluster analysis, it is inferred that mutations in *EPB41L4A* may be the cause of the associated clinical phenotypes in these patients. However, the findings from the clustering analyses require further validation through additional studies before drawing definitive conclusions.

*EPB41L4A* (erythrocyte protein band 4.1-like 4a, also named Nbl4) is a member of the FERM (4.1, Ezrin, Radixin, Moesin) protein superfamily, specifically the 4.1/Nbl4 (novel band 4.1-like protein 4) group. The proteins encoded by this gene family are involved in various cellular processes, including epithelial cell organization and signal transduction. At the molecular level, Band 4.1/Nbl4 proteins are known to link membrane-associated proteins and lipids to the actin cytoskeleton [28]. In this study, we identified that all six patients with unexplained tooth agenesis carried a splice acceptor mutation in the *EPB41L4A* gene (rs145708081, NM_022140.5:c.1933-6_1933–2dup). There is limited research on the role of the *EPB41L4A* gene, with studies primarily focusing on its involvement in blood glucose regulation, neurodegenerative diseases, and cancers such as thyroid, breast, and nasopharyngeal carcinomas. Y. Guo et al. confirmed that *EPB41L4A* is strongly and specifically expressed in the developing body, particularly during the segmentation process, as well as in structures such as the nose, cranial skeletal scaffolds, pronephros, and neural tube. They found that *EPB41L4A* is an important regulatory factor in embryonic development. Its expression in these critical areas suggests that it plays a significant role in the development and differentiation of various tissues and organs during early stages of development [29]. P. Cui et al. discovered that *EPB41L4A* can inhibit the proliferation of bone marrow-derived mesenchymal stem cells by interacting with microRNAs [30]. Meanwhile, Ishiguro et al. performed experiments using both mouse and human cells, and their comprehensive results showed that *EPB41L4A* is a target gene of the Wnt/β-catenin signaling pathway [31]. Further genetic studies revealed that the Wnt and Wnt-related pathways play a crucial role in the genetic etiology of tooth agenesis [32]. Therefore, the biological significance of *EPB41L4A* aligns well with the conclusions drawn from clustering analysis. The gene *EPB41L4A* may be an important risk factor associated with the development of tooth agenesis.

In biological‑sequence research concerning tooth agenesis, our study performs analyses based on mRNA sequences. Nucleic‑acid‑sequence analysis represents the initial step for sequence‑related exploration. Although amino‑acid‑level investigation is theoretically more reliable for evaluating protein function, we only carry out preliminary correlation analysis of variant loci at the nucleotide‑sequence stage owing to our current experimental constraints. Subsequent verification based on amino‑acid sequences for protein‑structure analysis will be conducted in our follow‑up research when conditions permit.

Clustering is a crucial data mining tool and an algorithm for separating data of similar nature. Unlike classification algorithms, clustering algorithms are unsupervised [13]. There are numerous clustering algorithms to choose from, but there is no single optimal clustering algorithm for all situations. To address the differences between various clustering analyses, both hierarchical clustering and fuzzy clustering methods were employed for clustering analysis. The consistent clustering results obtained from these different methods support gene functional classification and provide a basis for further uncovering the biological knowledge and patterns inherent in biological sequences.

## 5. Conclusion

In conclusion, we analyzed genes related to maxillofacial development shared among six patients with tooth agenesis of unknown etiology using two clustering analyses. Through this analysis, we identified a potential risk factor associated with tooth agenesis: *EPB41L4A*. However, the findings from the clustering analyses require further validation through additional studies before drawing definitive conclusions. This study provides a preliminary genetic reference that may contribute to the prevention and clinical diagnosis and treatment of tooth agenesis.

## References

[1] Song S, Zhao R, He H, Zhang J, Feng H, Lin L. WNT10A variants are associated with non-syndromic tooth agenesis in the general population. Hum Genet. 2014;133(1):117–24. doi: 10.1007/s00439-013-1360-x 24043634

[2] Zhang J, Liu HC, Lyu X, Shen GH, Deng XX, Li WR, et al. Prevalence of tooth agenesis in adolescent Chinese populations with or without orthodontics. Chin J Dent Res. 2015;18(1):59–65. 25815384

[3] Kanchanasevee C, Chantarangsu S, Pittayapat P, Porntaveetus T. Patterns of nonsyndromic tooth agenesis and sexual dimorphism. BMC Oral Health. 2023;23(1):37. doi: 10.1186/s12903-023-02753-1 36691053 PMC9869554

[4] Marra PM, Iorio B, Itro A, Santoro R, Itro A. Association of tooth agenesis with dental anomalies in young subjects. Oral Maxillofac Surg. 2021;25(1):35–9. doi: 10.1007/s10006-020-00879-y 32676748

[5] Bonczek O, Krejci P, Izakovicova-Holla L, Cernochova P, Kiss I, Vojtesek B. Tooth agenesis: What do we know and is there a connection to cancer?. Clin Genet. 2021;99(4):493–502. doi: 10.1111/cge.13892 33249565

[6] Vastardis H. The genetics of human tooth agenesis: new discoveries for understanding dental anomalies. Am J Orthod Dentofacial Orthop. 2000;117(6):650–6. doi: 10.1016/s0889-5406(00)70173-9 10842107

[7] Lammi L, Arte S, Somer M, Jarvinen H, Lahermo P, Thesleff I, et al. Mutations in AXIN2 cause familial tooth agenesis and predispose to colorectal cancer. Am J Hum Genet. 2004;74(5):1043–50. doi: 10.1086/386293 15042511 PMC1181967

[8] Song S, Han D, Qu H, Gong Y, Wu H, Zhang X, et al. EDA gene mutations underlie non-syndromic oligodontia. J Dent Res. 2009;88(2):126–31. doi: 10.1177/0022034508328627 19278982 PMC3317984

[9] Vastardis H, Karimbux N, Guthua SW, Seidman JG, Seidman CE. A human MSX1 homeodomain missense mutation causes selective tooth agenesis. Nat Genet. 1996;13(4):417–21. doi: 10.1038/ng0896-417 8696335

[10] Stockton DW, Das P, Goldenberg M, D’Souza RN, Patel PI. Mutation of PAX9 is associated with oligodontia. Nat Genet. 2000;24(1):18–9. doi: 10.1038/71634 10615120

[11] Arte S, Parmanen S, Pirinen S, Alaluusua S, Nieminen P. Candidate gene analysis of tooth agenesis identifies novel mutations in six genes and suggests significant role for WNT and EDA signaling and allele combinations. PLoS One. 2013;8(8):e73705. doi: 10.1371/journal.pone.0073705 23991204 PMC3750013

[12] Sangkaew S, Tan LK, Ng LC, Ferguson NM, Dorigatti I. Using cluster analysis to reconstruct dengue exposure patterns from cross-sectional serological studies in Singapore. Parasit Vectors. 2020;13(1):32. doi: 10.1186/s13071-020-3898-5 31952539 PMC6969465

[13] Jung YG, Kang MS, Heo J. Clustering performance comparison using K-means and expectation maximization algorithms. Biotechnol Biotechnol Equip. 2014;28(sup1):S44–8. doi: 10.1080/13102818.2014.949045 26019610 PMC4433949

[14] Dembélé D, Kastner P. Fuzzy C-means method for clustering microarray data. Bioinformatics. 2003;19(8):973–80. doi: 10.1093/bioinformatics/btg119 12761060

[15] Paranjyothi MV, Kumaraswamy KL, Begum LF, Manjunath K, Litha, Basheer S. Tooth agenesis: A susceptible indicator for colorectal cancer?. J Cancer Res Ther. 2018;14(3):527–31. doi: 10.4103/0973-1482.168997 29893310

[16] Date Y, Kondo H, Yamashita A, Iseki S, Kasugai S, Ota MS. Combined in silico analysis identified a putative tooth root formation-related gene, Chd3, which regulates DNA synthesis in HERS01a cells. Odontology. 2020;108(3):386–95. doi: 10.1007/s10266-020-00489-w 32026140

[17] Assiry AA, Albalawi AM, Zafar MS, Khan SD, Ullah A, Almatrafi A, et al. KMT2C, a histone methyltransferase, is mutated in a family segregating non-syndromic primary failure of tooth eruption. Sci Rep. 2019;9(1):16469. doi: 10.1038/s41598-019-52935-7 31712638 PMC6848163

[18] Kamiunten T, Ideno H, Shimada A, Nakamura Y, Kimura H, Nakashima K, et al. Coordinated expression of H3K9 histone methyltransferases during tooth development in mice. Histochem Cell Biol. 2015;143(3):259–66. doi: 10.1007/s00418-014-1284-0 25294562

[19] Froment C, Zanolli C, Hourset M, Mouton-Barbosa E, Moreira A, Burlet-Schiltz O, et al. Protein sequence comparison of human and non-human primate tooth proteomes. J Proteomics. 2021;231:104045. doi: 10.1016/j.jprot.2020.104045 33189847

[20] Glinton KE, Hurst ACE, Bowling KM, Cristian I, Haynes D, Adstamongkonkul D, et al. Phenotypic expansion of the BPTF-related neurodevelopmental disorder with dysmorphic facies and distal limb anomalies. Am J Med Genet A. 2021;185(5):1366–78. doi: 10.1002/ajmg.a.62102 33522091 PMC8048530

[21] Harms FL, Girisha KM, Hardigan AA, Kortüm F, Shukla A, Alawi M, et al. Mutations in EBF3 Disturb Transcriptional Profiles and Cause Intellectual Disability, Ataxia, and Facial Dysmorphism. Am J Hum Genet. 2017;100(1):117–27. 28017373 10.1016/j.ajhg.2016.11.012 PMC5223027

[22] Ansar M, Chung H-L, Al-Otaibi A, Elagabani MN, Ravenscroft TA, Paracha SA, et al. Bi-allelic Variants in IQSEC1 Cause Intellectual Disability, Developmental Delay, and Short Stature. Am J Hum Genet. 2019;105(5):907–20. doi: 10.1016/j.ajhg.2019.09.013 31607425 PMC6848997

[23] Rönkkö J, Molchanova S, Revah-Politi A, Pereira EM, Auranen M, Toppila J, et al. Dominant mutations in ITPR3 cause Charcot-Marie-Tooth disease. Ann Clin Transl Neurol. 2020;7(10):1962–72. doi: 10.1002/acn3.51190 32949214 PMC7545616

[24] Chauveau C, Rowell J, Ferreiro A. A rising titan: TTN review and mutation update. Hum Mutat. 2014;35(9):1046–59. doi: 10.1002/humu.22611 24980681

[25] Chang M, Lin H, Luo M, Wang J, Han G. Integrated miRNA and mRNA expression profiling of tension force-induced bone formation in periodontal ligament cells. In Vitro Cell Dev Biol Anim. 2015;51(8):797–807. doi: 10.1007/s11626-015-9892-0 26091625

[26] Li H, Yang HH, Sun ZG, Tang HB, Min JK. Whole-transcriptome sequencing of knee joint cartilage from osteoarthritis patients. Bone Joint Res. 2019;8(7):290–303. doi: 10.1302/2046-3758.87.BJR-2018-0297.R1 31463037 PMC6691371

[27] Wang X, Chen Y, Jin J, Zhang B. Fuzzy-clustering and fuzzy network based interpretable fuzzy model for prediction. Sci Rep. 2022;12(1):16279. doi: 10.1038/s41598-022-20015-y 36175517 PMC9523042

[28] Zhang W, Lai R, He X, Liu X, Zhang Y, Yang Z, et al. Clinical prognostic implications of EPB41L4A expression in multiple myeloma. J Cancer. 2020;11(3):619–29. doi: 10.7150/jca.33805 31942185 PMC6959044

[29] Guo Y, Christine KS, Conlon F, Gessert S, Kühl M. Expression analysis of epb41l4a during Xenopus laevis embryogenesis. Dev Genes Evol. 2011;221(2):113–9. doi: 10.1007/s00427-011-0362-8 21556855 PMC3612984

[30] Cui P, Zhao X, Liu J, Chen X, Gao Y, Tao K, et al. miR-146a interacting with lncRNA EPB41L4A-AS1 and lncRNA SNHG7 inhibits proliferation of bone marrow-derived mesenchymal stem cells. J Cell Physiol. 2020;235(4):3292–308. doi: 10.1002/jcp.29217 31612476 PMC13482691

[31] Ishiguro H, Furukawa Y, Daigo Y, Miyoshi Y, Nagasawa Y, Nishiwaki T, et al. Isolation and characterization of human NBL4, a gene involved in the beta-catenin/tcf signaling pathway. Jpn J Cancer Res. 2000;91(6):597–603. doi: 10.1111/j.1349-7006.2000.tb00987.x 10874211 PMC5926395

[32] Yu M, Wong S-W, Han D, Cai T. Genetic analysis: Wnt and other pathways in nonsyndromic tooth agenesis. Oral Dis. 2019;25(3):646–51. doi: 10.1111/odi.12931 29969831 PMC6318069

